# Development and Characterization of PVA/KGM-Based Bioactive Films Incorporating Natural Extracts and Thyme Oil

**DOI:** 10.3390/polym17172425

**Published:** 2025-09-08

**Authors:** Ayşenur Yeşilyurt

**Affiliations:** Department of Chemical Engineering, Faculty of Engineering and Natural Sciences, Bursa Technical University, Bursa 16310, Turkey; aysenur.erdogan@btu.edu.tr

**Keywords:** anthocyanin, antioxidant activity, betacyanin, natural extracts, PVA/KGM biocomposites, thyme oil

## Abstract

This study focused on the development and characterization of polyvinyl alcohol (PVA)- and konjac glucomannan (KGM)-based composite films enriched with natural bioactive additives. A PK (PVA/KGM) matrix with the optimum tensile strength was selected, and five film formulations were prepared by incorporating *Aronia melanocarpa* extract (AME), red dragon fruit extract (DFE), and thyme essential oil (TEO). TEO was also introduced via a Pickering emulsion (PE) technique. The total phenolic content (TPC) and free radical scavenging activity (FRSA) of extracts and films were determined, where AME exhibited the highest antioxidant activity (TPC: 243 mg GAE/g; FRSA: 81.7%). The additive-free PK film displayed limited antioxidant activity (18%), while antioxidant capacity significantly improved with extract and EO incorporation. The PK-A film (AME-added) demonstrated the highest tensile strength and lowest water vapor permeability, supported by increased local crystallinity detected in XRD. Color analysis indicated dominant red-violet tones in AME films and greenish-yellow tones in DFE films. FTIR confirmed that no new chemical bonds were formed between active compounds and the polymer matrix. DSC thermograms revealed consistent melting peaks (~150 °C) for all films, while Tg varied from 37 to 73 °C depending on additive type, reflecting plasticization effects of extracts and the counterbalancing effect of essential oil. The most hydrophobic (76.8°) and opaque sample was PK-ADO, prepared via the PE technique. Overall, natural extracts improved the structural, thermal, barrier, and antioxidant properties of PK films.

## 1. Introduction

Packaging—widely used especially in the food sector—ensures safe delivery, product durability, and convenience in handling/storage [1,2]. Conventional plastic packaging (PET, PVC, PP, PLA) protects against physical, chemical, and biological factors, facilitates transport, and supports marketing [3,4]. Yet their persistence leads to microplastics that threaten food safety and human health; particles detected in the gastrointestinal tract may disrupt lipid, protein, glucose, iron, and energy metabolism, and they have been found in human blood and tissues [5,6,7,8]. Sound risk assessment requires exposure levels and effects; reported concentrations exist for drinking water, tea bags, seafood, salt, and air [9]. For example, PET-bottled water averages ~14 particles L^−1^, and a Canadian study estimated ~16 μg migrating from tea bag packaging into hot water [10,11]. The COVID-19 period further increased single-use plastics, exacerbating waste [1,6]. Plastic packaging also elevates carbon emissions and energy use, releases harmful toxins, and damages marine ecosystems, generating economic losses (fisheries, tourism) and substantial cleanup/landfill costs [12,13,14].

The need to curb plastic use and develop sustainable food packaging has prompted research into eco-friendly, biodegradable materials. Biodegradable plastics are those that microorganisms can break down. They are broadly classified as (i) bioplastics from renewable feedstocks and (ii) petrochemical-based plastics containing additives that promote biodegradation [1,15]. Representative examples include polyvinyl alcohol (PVA), polyhydroxyalkanoates (PHA), polylactic acid (PLA), and polyglycolic acid (PGA) [16].

PVA is considered a viable alternative to petroleum-based plastics due to its strong physical performance [17]. It is an odorless, non-toxic, water-soluble, flexible, and transparent film-forming polymer with high tensile strength and chemical resistance; PVA-based films readily dissolve in water and have been increasingly used in food packaging and biopolymer films. Many studies show that combining PVA with various biological or chemical additives can enhance food safety, extend shelf life, and aid preservation [18,19,20]. However, limited water resistance and modest biodegradability remain challenges for packaging; therefore, PVA is often blended with inexpensive, readily available, biodegradable macromolecules such as starch, proteins, and polysaccharides to improve barrier and environmental performance [19,21].

Konjac glucomannan (KGM) is a hydrophilic, natural polysaccharide obtained from the underground tubers of Amorphophallus konjac and used in the chemical, pharmaceutical, medical, and food industries [12,19,22,23]. As a water-soluble dietary fiber it supports digestive health and intestinal motility [24], its low-calorie nature promotes satiety [12], and it has additional benefits such as glycemic control, cholesterol lowering, and potential cancer-risk reduction [24,25]. In foods KGM functions as an adhesive/thickener; being biodegradable, it is promising for packaging applications [19,22,26], and KGM-based packaging can suppress microbial growth and help preserve contents [27]. In PVA/KGM blends, the weaknesses of each component are mitigated: KGM’s poor mechanics and PVA’s limited biodegradability are reduced, and KGM’s abundant hydroxyls increase PVA’s water resistance to some extent, improving moisture tolerance versus neat PVA, although PVA/KGM films still tend to dissolve in water [16].

Food packaging serves four core functions: protection from physical, chemical, and biological factors; containment and safe transport; communication/information; and convenience in handling and use [3]. Smart packaging aims not only to enhance stability, quality, shelf life, functionality, and safety but also to report product status. Within this concept, active packaging interacts with food via functional agents (e.g., antimicrobials, antioxidants), whereas intelligent packaging uses indicators/sensors to monitor changes such as freshness or pH without direct contact [28]. Biodegradable polymers such as PVA, PLA, PHAs, and PGA are increasingly used; under microbial action their carbon backbones are metabolized into simple end-products (CO_2_, CH_4_) and biomass, consistent with natural carbon and energy cycles [16]. Enrichment with natural bioactives (vitamins, polyphenols, essential oils, plant extracts, amino acids) further supports health and sustainability. Natural pigments (anthocyanins, betalains) from blueberry, yerba mate, mulberry, purple sweet potato, black plum peel, and essential oils such as Cinnamomum zeylanicum, Niaouli, Cymbopogon flexuosus, cinnamaldehyde, and Alpinia galanga have improved the performance of smart indicators that monitor spoilage [16,29,30,31,32,33,34,35,36]. Such combinations enhance food safety and can also promote faster environmental breakdown of the packaging [34,37].

In recent years, increasing demand for bioactive compounds has stimulated research on different extraction techniques from plant-based sources, including conventional solvent extraction as well as ultrasonic- and microwave-assisted methods. The primary aim of these approaches is to retain the functional integrity of the bioactive molecules while at the same time reducing or avoiding the use of organic solvents, thus making the process more sustainable [31]. Jang and Koh [38] compared different methods for obtaining extracts in their studies. It was reported that there was no significant difference between the solvent extraction method with water and the other methods. This is not surprising considering the fact that anthocyanins are water soluble. [39]. However, Delgado-Vargas et al. [40] added that in most cases, organic solvents such as methanol or ethanol should be used to complete the extraction.

Aronia melanocarpa (AM; black chokeberry) is a Rosaceae shrub from temperate North America and Europe, often found in wetlands and along riverbanks [41,42]. It is rich in polyphenols—especially anthocyanins (e.g., cyanidin glycosides) and flavonoids such as (+)-catechin, (−)-epicatechin, and procyanidin B1—and also contains hydroxycinnamic (e.g., caffeic) and organic acids (e.g., citric) [43,44]. The high anthocyanin content confers strong antioxidant capacity [41,42,45], and is linked to reduced oxidative damage with anti-inflammatory and antidiabetic effects relevant to cardiometabolic health [4,38,46,47]. In the study conducted by Flint et al. [46], it was reported that Aronia melanocarpa extracts (AME) with citric acid added increased the efficiency of dye-sensitized solar cells by 4%. AM attracts attention with its environmentally friendly properties as well as its health benefits. For sustainable packaging, AM extracts function as natural colorants/preservatives with antimicrobial and antifungal activity, helping limit spoilage and extend shelf life [42,48].

Dragon fruit (DF; pitaya) is a tropical cactus fruit (Cactaceae) cultivated in regions such as the United States, Australia, and Mexico; red, yellow, and white-fleshed varieties exist. The peel accounts for roughly one-third of the fruit mass [49]. DF (Hylocereus polyrhizus) has gained interest for food and health applications: its peel contains vitamin C, water-soluble betalains (betaxanthins, betacyanins), polyphenols with antimicrobial/antioxidant activity, and unsaturated fatty acids (linoleic, linolenic) associated with immune and skin benefits [18,37,50,51]. Discarded peels are also rich in pectin and dietary fibers—natural polysaccharides suitable for food processing and packaging uses [49,52]. These constituents enhance antioxidant capacity, while DF flavonoids and phenolic acids contribute anti-inflammatory and antimicrobial effects [18].

Essential oils (EOs) are volatile, hydrophobic plant metabolites rich in terpenoids, phenols, and flavonoids, exhibiting antioxidant, antimicrobial, antidiabetic, and anti-inflammatory activities [53,54]. EOs from lemongrass, clove, cinnamon, tea tree, ginger, and rosemary are used as natural preservatives in health, cosmetics, and food packaging [53,54,55]. Incorporating thyme EO improves films’ antimicrobial performance and increases hydrophobicity [56,57]. However, directly adding EOs to the hydrophilic PVA/KGM matrix often yields non-homogeneous structures. Pickering emulsions—stabilized by solid particles—provide a surfactant-free, environmentally friendly way to disperse EOs. Cyclodextrins (CDs), starch-derived cyclic oligosaccharides, form inclusion complexes with diverse guests; notably β-cyclodextrin complexes lipophilic EOs, enhancing their solubility, stability, and bioavailability [54,58,59,60,61].

In this study, biodegradable and compostable PVA/KGM-based biopolymers will be combined with natural fruit (AME and DFE) extracts. AME and DFE have antibacterial and antioxidant effects due to their high anthocyanin and betalain content, while their natural coloring properties will increase the visual appeal of food products and add color to packaging systems [37,50,52,62]. These packages can change color according to the acidic or basic conditions around the food thanks to their natural pH indicator properties, thus helping to detect spoiled or deteriorated products early [63]. The effect of the combination of both compounds will also be investigated in the study. In addition to this effect, PVA/KGM biodegradable films loaded with thyme essential oil (TEO) will be produced with the PE methodology. The use of β-CD molecules to obtain PE is a very promising approach to stabilize emulsions. In addition, supporting the films with TEOs will provide the support of the film with antibacterial and healing properties, filling an important gap in the literature. The films to be produced will be examined in terms of morphological, physical, chemical, and biological efficiency.

## 2. Materials and Methods

### 2.1. Materials

High-molecular-weight PVA granules were supplied by Sigma-Aldrich (Burlington, MA, USA) (Mw: 89,000–98,000, 99% hydrolyzed), Chemie, Steinheim, Germany. Food-grade Konjac glucomannan (100% purity) was supplied by Tito, İstanbul, Türkiye. Glycerol, citric acid, and sodium carbonate were supplied by Merck (Darmstadt, Germany), β-cyclodextrin (β-CD), Folin–Ciocalteu reagent, 2,2-diphenyl-1-picrylhydrazyl (DPPH) were supplied by AFG Scientific. All chemicals were used as received. Thyme spice, AM, and DF powder were purchased from a local market in Türkiye.

### 2.2. Methods

#### 2.2.1. Extraction of Bioactive Compounds from Aronia Melanocarpa and Dragon Fruit

**Figure 1:**
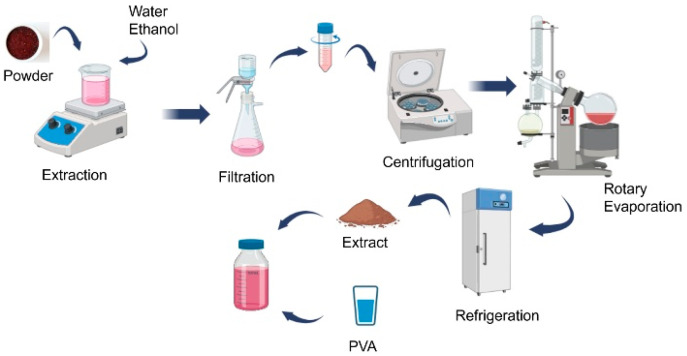
Extraction of bioactive compounds from *Aronia melanocarpa* and dragon fruit.

AM and DF powders were extracted with minor modifications to the method of Oun et al. [4]. 20 g of powder was added to 300 mL (90:10 *v*/*v* pure water: ethanol) solution. After 10 min of sonication, the mixture was stirred overnight at 40 °C. The resulting mixture was filtered through a vacuum apparatus and centrifuged at 3000 rpm for 30 min. After the insoluble materials were separated, the remaining liquid was removed in the rotary evaporator to obtain bioactive components. When not in use, the powders were stored in light-proof containers and in the freezer. The bioactive component extraction is given in Figure 1.

#### 2.2.2. Characterization of Bioactive Components of Aronia Melanocarpa and Dragon Fruit

##### Color Changes in Extracts at Different pHs

The color changes in both extracts at pH 4, 7 and 10 (acidic, neutral and basic) values were observed using buffer solutions following the method of Oun et al. [4]. For this purpose, 1 mL of the extract solution (5 mg/mL) was added to 1 mL of pH solutions. The resulting mixtures were recorded with a digital camera.

##### Total Phenolic Content (TPC) of Extracts

Total phenolic compound content of extracts was determined by the Folin–Ciocalteu test, with slight modifications to the method described by Lu et al. [64]. Pure water was evaluated as a blank sample. 1 μL (10 mg/mL) extract or pure water was added to 7.5 μL Folin–Ciocalteu (10% *v*/*v*) reagent. The mixture was incubated at room temperature for 10 min. Then, 3 μL sodium carbonate solution (5% *v*/*v*) was added to the mixture and incubated for another 60 min at room temperature. The absorbance of the mixture was measured at 760 nm on a HITACHI/U-4100 brand/model UV spectrometer. The results were measured in the gallic acid calibration curve and presented as mg gallic acid equivalent (GAE)/g extract.

##### Determination of Antioxidant Activity of Extracts

The antioxidant activities of the extracts were determined by the DPPH radical scavenging method. Pure water was used for the blank sample. 0.2 mL DPPH was added to the extracts at different concentrations and incubated at room temperature for 60 min. The absorbance value of the mixture was recorded at 517 nm on a HITACHI/U-4100 brand/model UV spectrometer and free radical scavenging activity (%, FRSA) was calculated according to Equation (1).

(1) $$FRSA\left(\%\right)=\frac{A_{0}-A_{S}}{A_{0}}\;*\;100$$

In the calculation of free radical antioxidant activity (%), A_0_ refers to the absorbance value of the reference, and A_S_ refers to the absorbance value of the sample.

#### 2.2.3. Obtainment of Thyme Essential Oil

Obtainment of essential oil from thyme spice was carried out by solvent extraction method. For this purpose, 50 g of thyme powder was taken into the soxhlet extractor cartridge. Ethyl alcohol (250 mL) was selected as the extraction solvent. After the extraction process, which lasted approximately 10 h, ethyl alcohol was removed from the ethyl alcohol + oil mixture collected in the soxhlet flask by rotary evaporator. The obtained thyme essential oil was stored in a dark glass bottle in the refrigerator.

#### 2.2.4. Determination of the Components of Thyme Essential Oil

Determination of the components of thyme essential oil was carried out by Gas Chromatography/Mass Spectroscopy (Agilent 7890B / Agilent 5977B, Agilent Technologies, Santa Clara, CA, USA) device. The sample diluted with hexane was analyzed in the device using HP-5MS column (stationary phase) and Helium gas (mobile phase). The temperature program (waiting for 2 min at 60 °C, reaching 250 °C with an increase rate of 3 °C/min, waiting for 3 min at 250 °C) was applied for the analysis. The peaks in the chromatogram obtained after the analysis were matched in the NIST library.

#### 2.2.5. Determination of Optimum Ratio of PVA/KGM Films

In order to determine the PVA/KGM ratio with the best mechanical strength in biodegradable packaging film production, PVA/KGM (*v*/*v*, 90/10, 75/25, 50/50) films with different ratios were produced by solvent casting method.

Film production was started by preparing solutions of PVA granules and KGM powder in pure water. 10% (*w*/*v*) solution of PVA granules was prepared at 90 °C and 0.5% solution of KGM powder was prepared at 40 °C. After obtaining homogeneous solutions, different volumes of PVA/KGM solutions were mixed in a magnetic stirrer at 40 °C for 6 h. Citric acid and glycerol were added to the biodegradable film solutions in the amounts given in Table 1, respectively. Each component was mixed for a minimum of two hours. Thirty grams of film solutions were transferred to 12 cm Petri dishes. The films, whose solvents were removed at 30 °C, were stored in a cool environment.

Citric acid was added to the films as a cross-linking agent. Citric acid is a cheap, harmless compound with antibacterial properties against Gram-negative bacteria. Cross-linking occurs through the esterification reaction between heated citric acid (-COOH) and carbohydrate [65,66,67].

#### 2.2.6. Characterization of Different Ratio PVA/KGM Films

##### Mechanical Testing

The tensile strength, young modulus and elongation at break values of PVA/KGM films produced at different ratios were recorded according to the standard “ASTM D882-18: Standard Test Method for Tensile Properties of Thin Plastic Sheets” [68]. Before the measurements, the film samples were conditioned for 40 h at 23 °C and 50% relative humidity in accordance with Procedure A of “ASTM D618-21: Standard Practice for Conditioning Plastics for Testing” [69]. For the determination of mechanical properties; 1 kN capacity Testform/AS1, Ankara, Turkey was used. Biodegradable film samples prepared in 50 mm × 10 mm dimensions were measured at a constant deformation rate of 25 mm/min. Mechanical properties for each sample were evaluated with five different measurements at 50% relative humidity and room temperature and the obtained data were presented as mean ± standard deviations.

The obtained data were presented as mean ± standard deviations. Based on these results, further film studies were conducted using the PVA/KGM (*v*/*v*, 90/10) sample, which exhibited the best mechanical strength.

#### 2.2.7. Production of PVA/KGM-Based Biodegradable Films

**Figure 2:**
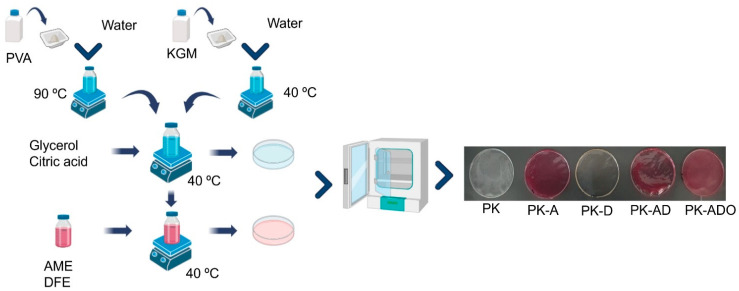
Film production scheme.

Citric acid and glycerol were added to PVA/KGM solutions with a volume ratio of 90/10 at two-hour intervals. AME and/or DFE 10% (*w*/*v*) solutions were added to the films containing extracts. For the EO loaded film, β-CD and TEO were added after the extracts, respectively. 30 g of the solutions mixed for 6 h were transferred to 12 cm Petri dishes using the solvent casting method. The films, the solvents of which were removed in an oven at 30 °C, were stored in a cool environment. The film without AME, DFE, or TEO was used as a reference sample. The reference and biodegradable film production scheme is given in Figure 2, and their compositions are given in Table 1.

#### 2.2.8. Characterization of PVA/KGM-Based Biodegradable Films

##### Fourier Transform Infrared (FT-IR) Spectroscopy

The chemical interactions and bond structures of the films obtained with a Perkin Elmer/Spectrum Two FT-IR spectrophotometer with diamond crystal were examined. The examination was carried out in transmission mode, with a resolution of 4 cm^−1^ and an average of 16 scans in the wavenumber range of 4000 to 400 cm^−1^.

##### Surface Morphology and Microstructural Features

The surface structures and cross-sectional morphologies of films were examined using a Carl Zeiss/Gemini 300 model Scanning Electron Microscope (SEM) (Oberkochen, Germany). In the SEM technique, the surfaces must be conductive in order for the electrons sent to the samples to be transmitted to the inner side of the sample. For this purpose, the surfaces of the biodegradable film samples were coated with a gold-palladium alloy metal target with a thickness of 15 nm using a Leica/ACE600 device (Wetzlar, Germany). Morphological measurements were taken at 100× (surface) and 250× (cross-section) magnification under 5 kV voltage.

##### Thickness Measurement and Mechanical Properties

The thickness of the produced films was measured at five different points on each biodegradable film sample using a digital caliper from Macrona, Turkey. The measured values were reported with their arithmetic means and standard deviations. The mechanical properties of biodegradable food packaging films were carried out as stated in the title “Determination of optimum ratio of PVA/KGM films”.

##### Color Measurements and Opacity

Color measurements and opacity measurements are important in order to protect packaged products from UV-VIS light radiation (which causes lipid oxidation of the packaging film, loss of flavor and nutrients). The color of the packaging film can also directly affect the appearance of the food and consumer acceptance, which are the basic parameters of the film [70,71].

Color measurement of plastic films was performed using Konica Minolta brand color spectrophotometer. D65 daylight colorimeter’s white color (L*: 97.34, a*: −0.03, b*: −0.12) kit was measured as a reference sample. In the measurements taken in the air environment, lightness (L), red/green (a / +,−) and yellow/blue (b / +,−) values of the samples were recorded by taking measurements from five different points of each sample. Each of the L, a, b values was given as mean ± standard deviation. The total color difference (deltaE) values of the film samples were calculated according to Equation (2). with the L, a, b values [72].

(2) $$∆E=\sqrt{{\left(L\;*-L\right)}^{2}+{\left(a\;*-a\right)}^{2}+{\left(b\;*-b\right)}^{2}}$$

The opacity values of the biodegradable film samples were calculated according to Equation (3). The absorbance values of the samples at 600 nm (Abs_600_) were measured with Hitachi U-1900 (brand/model, Tokyo, Japan) Ultraviolet-Visible spectrophotometer [72].

(3) $$Opacity=\frac{{Abs}_{600}}{X}$$

##### Surface Wettability and Hydrophilic Character

Contact angle measurements allow the determination of the interaction capacity of biodegradable films with water. The wettability properties of the films were examined using the Attension mark Theta Lite model (Biolin Scientific, Espoo, Finland) goniometer. Samples cut in 20 mm × 20 mm dimensions were tested with distilled water. The internal angle formed between the tangent drawn by the device and the drop at the point where the water drop contacted the film surface was recorded as the contact angle. The measurements were carried out under laboratory conditions.

##### Thermal Stability and Degradation Behavior

Thermogravimetric analysis (TGA, TA Instrument/SDT 50, New Castle, DE, USA) measurements were performed in order to determine the thermal degradation behaviors of biodegradable film samples in a wide temperature range. The measurements were taken in a nitrogen gas atmosphere between 25 °C and 600 °C. A constant temperature increase of 10 °C/min was applied for the measurements using alumina pans

##### Thermal Transition Properties

The thermal transition properties of the films were analyzed using a differential scanning calorimeter (DSC, TA Instruments, DSC250, New Castle, DE, USA). Approximately 10 mg of each sample was placed in an aluminum pan and subjected to three heating–cooling–heating cycles between –20 °C and 250 °C at a constant heating/cooling rate of 15 °C/min. An isothermal hold of 5 min at 250 °C was applied during the first heating cycle to erase thermal history. The data obtained from the third heating cycle were used for analysis of glass transition and melting behavior.

##### Crystalline–Amorphous Structure Analysis

The crystalline structure of the films was characterized by X-ray diffraction (XRD) using a Bruker D8 Advance diffractometer equipped with a Cu Kα radiation source (λ = 1.5406 Å) generated from a 1.8 kW copper anode tube. Measurements were carried out at 40 kV and 30 mA under continuous scanning mode. Diffraction patterns were collected in the 2θ range of 5–50° with a step size of 0.02° and a counting time of 1 s per step. The obtained diffraction patterns were used to evaluate the crystalline and amorphous domains of the PVA/KGM-based films and to assess the influence of natural extracts and essential oil incorporation on the overall crystallinity.

##### Antioxidant Activities

Aqueous solutions of films (10 mg/mL) were prepared. Each film solution, which was kept at room temperature overnight, was mixed with DPPH solution (1:1 *v*:*v*). Absorption values of samples kept in a light-free environment for 1 h were recorded. Antioxidant activities of film samples were calculated according to the equation titled “Determination of antioxidant activity of extracts” and blank measurements were taken against pure water.

##### Moisture Barrier: Water Vapor Permeability and Moisture Content

Water vapor permeability (WVP) of packaging films was tested according to the ASTM E96-24 “Standard Test Methods for Gravimetric Determination of Water Vapor Transmission Rate of Materials” standard method [73]. Film samples were cut into 4 cm × 4 cm dimensions and WVP containers were kept in a humidity chamber and the temperature and relative humidity were adjusted to 25 °C and 50%. Water vapor permeability values of the samples were calculated with Equation (4) [74].

(4) $$WVP(g/msPa)=\frac{WVTR\;*\;X}{\Delta Pa}$$

The water vapor transmission rate (WVTR) of the films is the slope of the weight loss-time graph recorded every 1 h for 8 h. X is the thickness of the samples and ΔPa is the water vapor partial pressure difference.

Film samples cut into 2 cm × 2 cm dimensions were kept at 105 ± 2 °C until they reached constant weight. The moisture content (MC, %) of the film samples was calculated using Equation (5) based on the dry mass before (W_1_) and after (W_2_) drying.

(5) $$MC(\%)=\frac{W_{1}{-W}_{2}}{W_{1}}\;*\;100$$

##### Biodegradation Performance in Soil Environment

A degradability test was applied in a controlled soil environment to evaluate the degradability of the film samples under environmental conditions. Each film was cut into approximately 2 cm × 2 cm dimensions and their initial dry mass (W_1_) was recorded. The cut film pieces were placed in pots filled with organic matter-rich and well-aerated natural soil at a depth of 5 cm. The pots were kept in the laboratory environment under constant humidity (50–60%) and temperature (approximately 25 °C) conditions for 30 days. At the end of the specified period, the film pieces were carefully removed from the soil, the surfaces were washed with pure water and dried, then dried at 105 ± 2 °C for 24 h and their final mass (W_2_) was measured. The degradability rate in soil (%) was calculated with Equation (6).

(6) $$SD\left(\%\right)=\frac{W_{1}{-W}_{2}}{W_{1}}\;*\;100$$

##### Statistical Analysis

The statistical evaluation of the data was carried out using the SPSS 22.0 package program. The significance of the differences observed between different film samples was determined by one-way analysis of variance (ANOVA), and the level of differences between the groups was evaluated according to the Tukey test. The results obtained were examined at a 95% confidence interval and the *p* < 0.05 value was considered statistically significant.

## 3. Results and Discussions

### 3.1. Characterization of Bioactive Compounds of Aronia Melanocarpa and Dragon Fruit Extracts

#### 3.1.1. pH Sensitivity of Extracts

**Figure 3:**
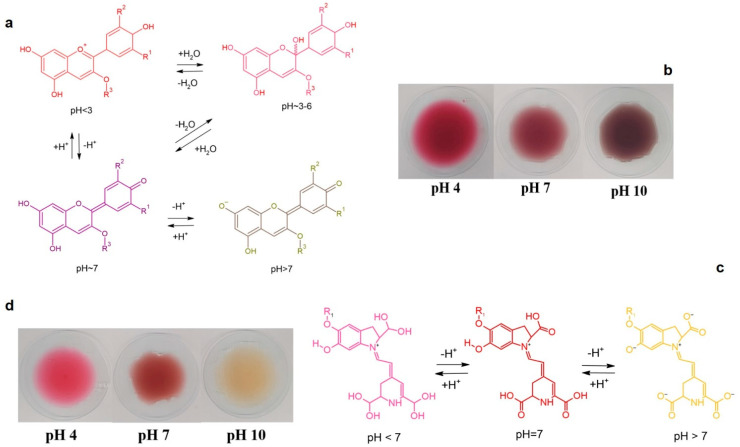
Molecular structures of (**a**). Anthocyanin at different pH values (**b**). Betacyanin at different pH values, Color changes in (**c**). AME (**d**). DFE at different pH values (4, 7, 10).

The molecular structures of anthocyanins and betacyanins at different pH values and the color changes in AME and DFE at different pH values (4, 7, 10) are given in Figure 3. AME and DFE show distinct color changes at different pH levels due to their pH-sensitive natural pigments. When the pH-dependent color change behavior of AME was examined (Figure 3a,b), distinct tone differences were observed according to the acidic, neutral or basic character of the medium. At pH 4 condition, the extract exhibited a light red color, and this appearance represents acidic environments where anthocyanins are dominant in the flavylium cation form. At neutral conditions (pH 7), the color of the solution turned purple; this change was associated with the formation of hydroxylated quinonoidal base forms in the molecular structure. In alkaline medium (pH 10), the color shifted to brown. The findings are consistent with the results of Oun et al. [4] The color transitions exhibited by dragon fruit extract in different pH values (Figure 3c,d) are due to the pH-sensitive structure of the betacyanin pigments it contains. In acidic environment (pH 4), the color of the extract was observed as bright red. This appearance can be attributed to the fact that betacyanins are stable and colored in acidic conditions. In this environment, the pigment structure maintains its stability and a vivid red color dominates the solution. When neutral conditions are passed (pH 7), the extract turned into a distinctly purplish tone. This change is due to the pigment structure changing to different ionic forms depending on the pH of the environment. In alkaline environment (pH 10), the color of the extract turned into yellow tones. This observation indicates that betacyanins begin to lose their stability in high pH conditions, their structures break down and turn into compounds that do not exhibit pigmentary properties. The findings are consistent with the results of Qin et al. [75].

Both extracts, which have a pH indicator feature, are suitable for evaluation as natural, environmentally friendly and visually stimulating biopigments for smart packaging applications, thanks to their distinct color changes at different pH levels.

#### 3.1.2. Total Phenolic Content of Extracts

Phenolic compounds constitute the main building blocks of natural antioxidants of plant origin and are important in many applications due to their biological activity potential. The TPC values of the extracts are similar to the literature data, but this value may vary depending on the fruit type, extraction conditions and solvent system used [4,51,76].

As a result of spectrophotometric measurements, AME exhibited a TPC value of 243 mg GAE/g extract. This value indicates that AME is a rich source of polyphenols (water-soluble phenolic compounds such as anthocyanins, flavonoids and tannins) [48]. Red pitaya fruit is a tropical fruit that attracts attention both in terms of color properties and functional bioactive components. As a result of the TPC analysis performed in this study, the total phenolic content of DFE was determined as 0.64 mg GAE/g extract. Nur et al. [51] extracted two dragon fruit species in different solvent media and reached the highest TPC value (79.00 ± 4.58 mg GAE/100 g sample) in the aqueous extraction medium. Since phenolic compounds are mostly directly related to antioxidant capacity, quantitative determination of such components is an important tool in determining the functional potential of natural resources. In this context, the obtained result shows that DF, especially its aqueous extracts, contain measurable levels of polyphenols and can provide moderate biological activity other than its colorant function. It should also be considered that the antioxidant capacity of DF may originate from other groups such as betalains other than polyphenolic compounds [77].

#### 3.1.3. Antioxidant Activities of Extracts

One of the widely preferred methods to determine the radical scavenging capacity of antioxidant compounds is spectrophotometric analysis using the DPPH radical. Since the DPPH molecule has an electron deficiency in its structure, it loses its color by reacting with antioxidants that can provide it with electrons or hydrogen, and this color change can be determined quantitatively at a wavelength of 517 nm.

**Figure 4:**
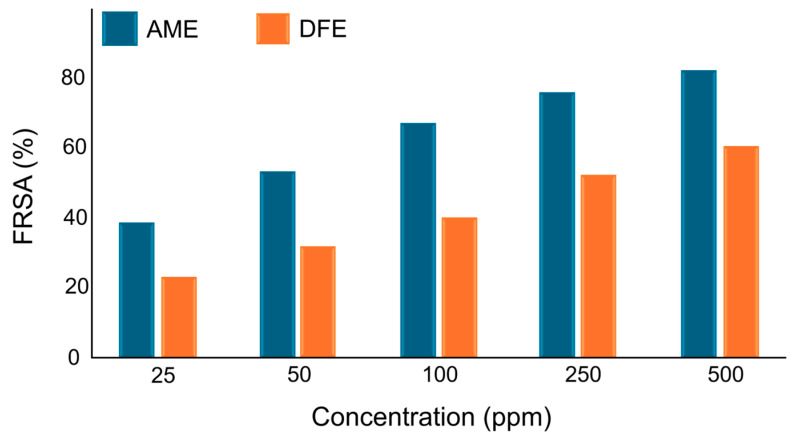
Free radical antioxidant activity (%) values of extracts depending on concentration.

The concentration-dependent expression of the free radical scavenging potentials of two different plant-based extracts is given in Figure 4.

AME and DFE exhibited significant antioxidant activity in the entire concentration range. AME reached over 80% inhibition rate at 500 µg/mL concentration. Both extracts showed significant radical scavenging capacity even at low concentrations. This finding shows that phenolic substances contained in AME can effectively neutralize free radicals and the extract has high biological activity [34,63]. Phenolic compounds found in DFE (0.64 mg GAE/g) were quite low compared to AME. However, although not as much as AME, it showed antioxidant activity at a level that could be used as food packaging material [75]. The antioxidant properties of DFE are explained by the antioxidant effects of betalain pigments and/or ascorbic acid [75,78]. These results are also consistent with the absence of anthocyanin and betacyanin derivatives. Although anthocyanins are found in different parts of the fruit, the anthocyanin content in red pitaya fruit is quite low [77].

In summary, AME exhibited a strong natural antioxidant profile by reaching both fast and high inhibition levels, while DFE showed a slower but supportive effect increasing with concentration.

### 3.2. Composition of Thyme Essential Oil

In the volatile component analysis of thyme essential oil, Thymol (10.9%), Carvacrol (85.4%) and other volatile compounds (3.7%) were detected.

### 3.3. Mechanical Performance-Based Determination of the Optimum PVA/KGM Ratio

Mechanical strength results of films produced at different ratios are given in Table 2. When Table 2 is examined, it is seen that the Young modulus and tensile strength values of the films decrease significantly as the KGM ratio increases. For this reason, studies were carried out on the 90/10 (*v*/*v*) sample for the PVA/KGM base with the best mechanical strength values within the scope of our study.

### 3.4. Characterization and Property Evaluation of PVA/KGM-Based Biodegradable Films

#### 3.4.1. Molecular Interactions and Structural Confirmation by FTIR

**Figure 5:**
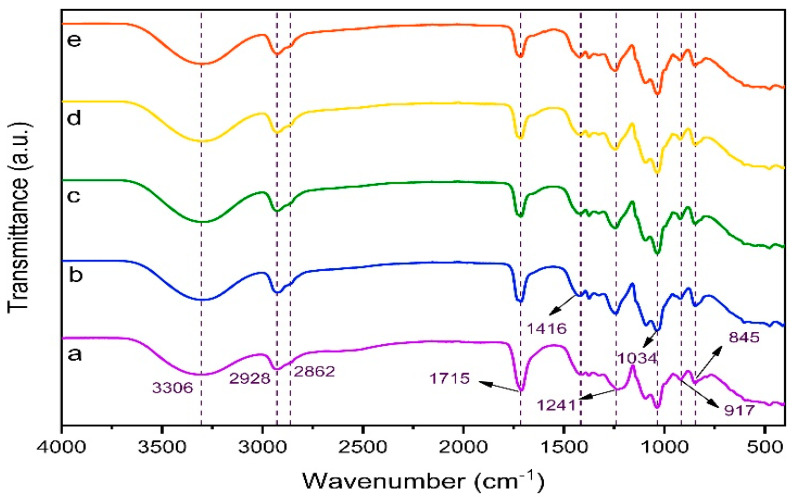
FTIR spectra of film samples (a: PK, b: PK−A, c: PK−D, d: PK−AD, e: PK−ADO).

FTIR spectra were taken to evaluate the bonding and functional structures of biodegradable films. In addition, chemical interactions of extracts and thyme oil added to the PVA/KGM blend polymer film were investigated with FTIR spectra. The peaks observed at 3306 cm^−1^, 2928 cm^−1^ and 2862 cm^−1^ in Figure 5a are attributed to –OH and asymmetric and symmetric –CH stretching peaks, respectively [12]. The peak at 1715 cm^−1^ is attributed to the esterification peak between PVA and KGM, and the peak at 1416 cm^−1^ is attributed to the aliphatic –CH stretching peak. The peaks at 1034 cm^−1^, 917 cm^−1^, and 845 cm^−1^ are attributed to the stretching vibration of the C–O–C glycosidic bond in the pyran ring, and the stretching vibration of the β-glycosidic and α-glycosidic bonds, respectively [79,80]. The peak located at 1233 cm^−1^ was attributed to the –CH stretching vibrations of the ester groups [66]. When Figure 5b–d and e were examined a different peak from Figure 5a, a new bond formation, was not observed. This showed that the added extracts and essential oils caused negligible changes in the chemical structures of the biodegradable films.

#### 3.4.2. Morphological Observations and Microstructural Interpretation

**Figure 6:**
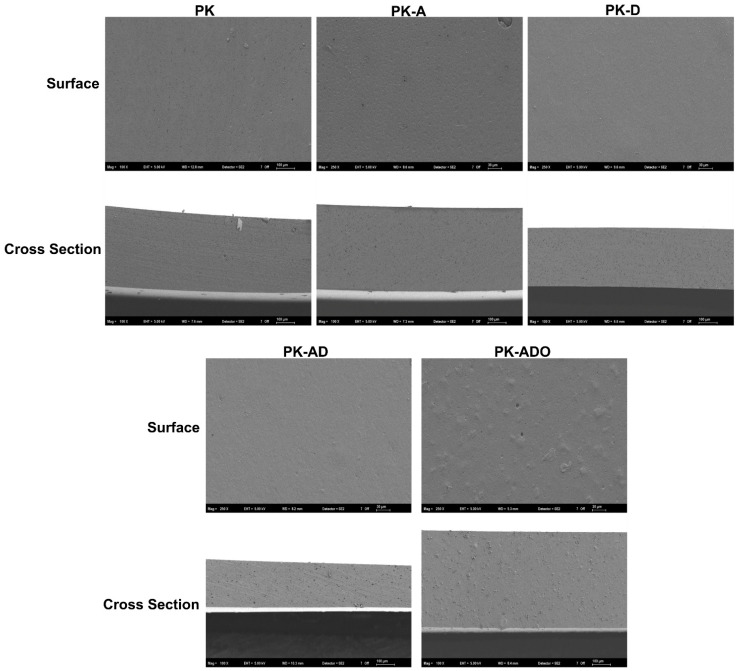
Surface and cross-sectional morphologies of the film samples.

The microstructures and surface morphologies of the produced composite film samples were examined with the SEM device (Figure 6).

In the images obtained from the cross-section and surface morphologies of the PVA/KGM reference film sample, it was seen that there were some holes on the surface. Liang et al. [35] recorded similar SEM images in PVA/KGM-based films. This is an expected situation in films containing high PVA ratios [42].

Micropores and holes continued to be observed in the surface and cross-section images obtained from the AME and DFE added film samples, but no phase separation was observed. The surface changes are explained by the formation of local densities during the dispersion of the extracts in the PK matrix and/or the evaporation of the solvent during the drying of the film. Liang et al., obtained similar images in the film samples when natural extracts were added to the PVA/KGM films. They reported that anthocyanins added to the polymer matrix disrupted the polymeric network structure of the film [35]. However, the morphological images of the film added to the AME and DFEs with thyme oil were more homogeneous. This situation can be explained by the formation of inclusion complexes by thyme oil and β-CD added in the aqueous solution. For PEs, EO concentration/β-CD and O/W (oil/water) ratios are important [61]. In the literature, it is reported that oil droplets are encapsulated in the biopolymer matrix in films using Pickering type emulsion and contribute to the smooth morphology. This structural integrity is also consistent with the high flexibility values observed in the film. The high EAB values observed especially in PK-D and PK-ADO films indicate that the microporous structure formed in the film allows the polymer chains to slide. The surface morphology and mechanical strength values are compatible.

#### 3.4.3. Film Thickness and Mechanical Performance Analysis

The hardness (Young Modulus-YM), flexibility (Elongation at Break-EAB) and maximum mechanical strength (Tensile strength-TS) values of the biodegradable packaging films were examined and presented in Table 3. TS value of the reference sample was recorded as 13.97 ± 1.52 MPa. With AME additive, tensile strength increased by 25.7% compared to PK film, which means that AME additive increased the strength of the polymer matrix. PK-D, PK-AD films had similar TS values with PK film. PK-ADO film exhibited similar properties to PK and PK-A films. This shows that extract/essential oils are compatible with the polymer matrix and can effectively transmit the stress applied on them. The ease of effective transfer of stress increased the resistance of the films against breaking force. The YM value of the PK film was 9.02 ± 0.24 MPa and an increase was observed in the YM values of all films except PK-AD (*p* < 0.05). The increase in the YM values of the PK-A, PK-D and PK-ADO films compared to the PK film was 75.94%, 29.9% and 28%, respectively. The sample with the highest YM value was PK-A. The high YM value indicates the resistance of the sample to deformation. The EAB value expressing flexibility was 538.69 ± 48.75% for the PK film. While AME decreased the flexibility of the film, DFE increased the flexibility of the film. AME contains abundant phenolic compounds; these compounds can stiffen the structure and restrict mobility by making hydrogen bonds with the polymer chains. The increase in the EAB value of the PK-D film can be explained by the plasticizing effect of DFE in the film [75]. There are no significant differences between PK, PK-AD and PK-ADO films and between PK-A and PK-D films. Oun et al. [4] observed an improvement in the mechanical properties of the polyvinyl alcohol/chitosan reference film due to good polymer/matrix interaction when aronia extract was added to it. Halasz et al. [48] reported that the EAB value of the chitosan film with aronia extract was reduced compared to the reference chitosan film due to the decrease in polymer chain movements. Addition of thyme essential oil to the PK-AD mixture increased the EAB value. In the studies, it was found that essential oil loading creates a heterogeneous structure in the composite due to oil-water phase separation and limits the movements of the matrix, which reduces TS and/or EAB values [81,82]. However, in recent years, the PE method has been used in the production of the film in order to harmonize the oil-water phase separation [83]. This situation is explained by the fact that thyme essential oil is compatible with polymer and bioactive components through emulsifier, creates a homogeneous mixture, increases the free movement of polymer chains and the elasticity value of the film due to the oil structure [83,84]. Liu et al. [81] added TEO to the KGM directly and by the PE method. While the mechanical values of the film with direct essential oil added decreased due to the oils disrupting the integrity of the film, the oil stabilized with PE created a more homogeneous structure in the film, and the KGM chain movements increased the EAB value.

The TS and EAB values of packaging films used for food preservation are particularly important in terms of being criteria expressing the stability of the films [80,84]. The TS and EAB values of all films (except PK-AD) are high compared to the reference film. While AME added hardness to the film sample, DFE and thyme oil increased flexibility and gave the polymer matrix a more elastic character.

#### 3.4.4. Colorimetric Response and Optical Behavior of Films

The L, a, b and calculated delta E values of plastic packaging materials are presented in Table 3. The L value, which expresses the degree of lightness/darkness, is defined with a value in the range of 100–0 [85,86]. The L value of the PVA/KGM film was recorded as 96.64. This value indicates the lightness of the film. The a and b values of the PK film were recorded as close to 0 and the deltaE value was recorded as 3.16. This value indicates that there was no visible change in the PK film. When AME was added to the PK film, the L and b values decreased significantly, while the a value increased significantly. This is consistent with the red color of AME [4,48]. The film became darker and the color showed a difference that could be easily detected by the human eye. This result shows that the anthocyanins contained in AME give a strong color to the film matrix and limit the light transmittance. In the digital images given in Figure 2, it is seen that the PK-A film has a red and dark color compared to the PK film. The L value remained high in the DFE-added film (96.43) and is very close to the reference film. This situation shows that the additive did not cause a darkening in the film color. However, the a value (−0.65) progressed in the negative direction and the b value in the positive direction (4.92). In other words, the film took on a slightly greenish-yellowish color. The ΔE value was 6.13, which is noticeable to the human eye, indicating that DFE creates a slight but perceptible color change. This effect can be explained by the betalain-derived pigments found in the fruit. PK-AD film, the L value (81.81) decreased, in other words, the color became darker but remained lighter than PK-A. The a value is quite high (24.77) and there is a red shift, but the b value approaches zero due to the effect of red pitaya, indicating that a red-purple tone is formed. The ΔE value of 30.53 represents a difference that is easily perceived by the eye. The color is dominated by the pigment density of AME, but the DFE contribution also contributed to the lightening of the tone. Indeed, in digital images, this film does not have as dark a red color as the PK-A coded sample. The L value of the PK-ADO film (74.09) is one of the lowest, and the film is quite dark. Since the a and b values are positive, the tone has shifted to the red-yellow range. The ΔE value shows that the total color difference in the film is very pronounced. The results are in accordance with Figure 2.

The light transmittance of the film samples was evaluated through opacity values and the results obtained provided information on the visual transmittance of the film matrix. The reference film (PK) exhibited a medium level of light transmittance due to the natural structure of the matrix with an opacity value of 3.67 units. This value shows that PK films without additives are semi-transparent and can transmit light at a basic level. The opacity value of the PK-A film is 4.83. It is thought that intensely colored compounds such as anthocyanin cause less light transmission in the film structure. At the same time, the fact that such phenolic compounds are more densely distributed in the film matrix and create a light scattering effect may also be effective in increasing opacity [81,87]. PK-D has a higher opacity value than PK film due to the colored components in it. The opacity value of PK-AD film is between the opacity values of PK-A and PK-D films. The sample with the highest opacity is PK-ADO film. PEs reduces light transmittance by refracting and dispersing the light beam entering the polymer matrix. In this case, high opacity results and increases the preservation of the product in the package [83].

#### 3.4.5. Wettability and Surface Hydrophilicity Assessment of Films

**Figure 7:**
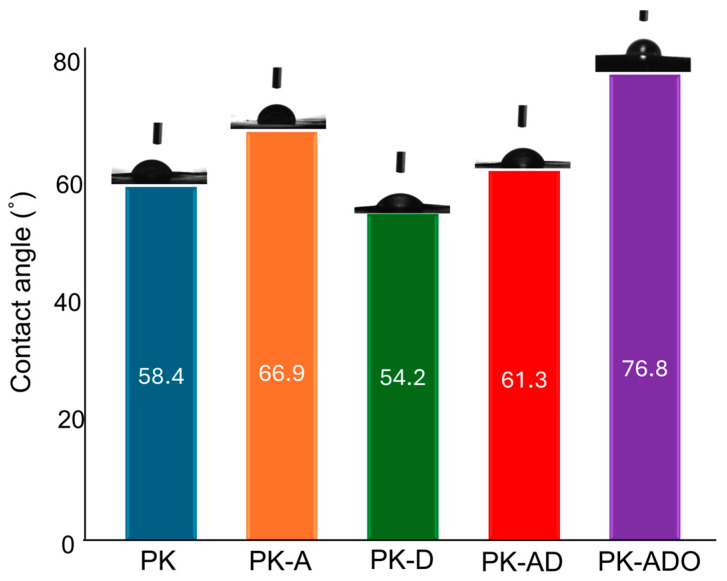
Contact angle measurements of neat and composite films.

The wettability behavior of the film samples was evaluated by water contact angle measurements (Figure 7). A contact angle value greater than 65° indicates that the surface is hydrophobic [86,88].

The reference film showed a slightly hydrophilic surface feature with a contact angle value of 58.4°. This value is consistent with the structural characteristics of hydrophilic polymers such as PVA and KGM and shows that it can be compatible with water-based systems [81]. The contact angle value reached 66.9° with the addition of AME to the reference film. This increase in the contact angle value shows that the water repellency of the surface increases with the integration of polyphenols in the extract into the structure. The aromatic structures of phenolic compounds in this film, which interact less with water, can be explained by the less wetting of the surface. The contact angle in the film sample with DFE addition showed the lowest value with 54.2°. The contact angle value of the PK-AD film sample is in the range of the contact angle values of PK-A and PK-D films with 61.3°. The film prepared with the addition of thyme oil reached the highest contact angle value (76.8°) and formed a clearly hydrophobic surface. Essential oils stabilized with PEs or homogeneously distributed in the film structure can increase the contact angle by closing the gaps in the polymer matrix and increasing the density of hydrophobic structures on the surface [54].

#### 3.4.6. Thermal Degradation Profile of Films

**Figure 8:**
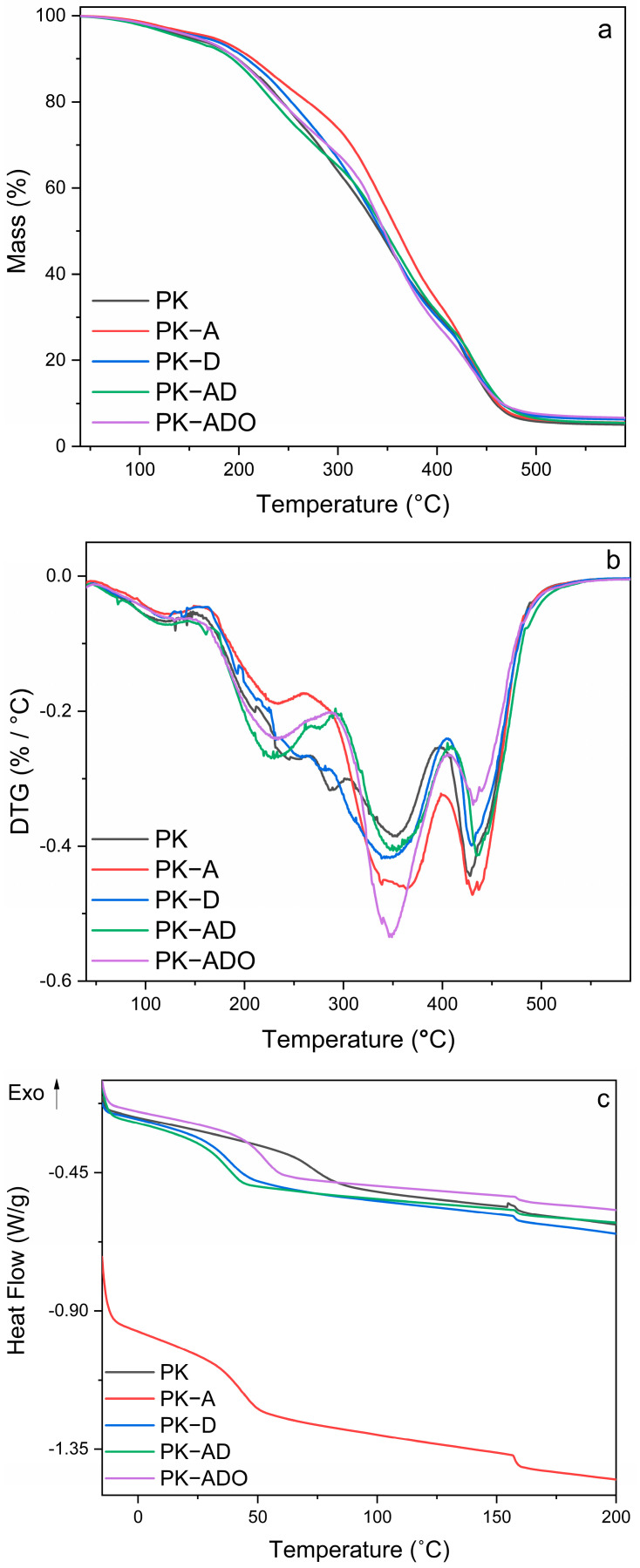
Thermogravimetric (**a**), derivative thermogravimetric (**b**) and differential scanning calorimetry (**c**) curves of the prepared film samples.

Thermal degradation behaviors of five different films produced were evaluated by TGA analysis. Thermogram and DTG (a derivative of TGA) curves are given in Figure 8. Four main degradation stages were observed in each film. Physical water and low-molecular-weight volatile compounds held in the film matrix were removed in the range of 50–150 °C [63]. The mass loss observed in the range of 160–250 °C was associated with the degradation of glycerol, which is present as a plasticizer in the film. When glycerol is heated, it separates from the film by undergoing dehydration and decomposition processes [27].

The third degradation step is due to the basic degradation of KGM [19]. The third and fourth degradation steps are mainly due to depolymerization of the main and side chains of polyvinyl alcohol and glucose dissolution of β-CD [72]. When the degradation start and end temperature values of the films were examined, the thermal stability of all films except the PK-D film increased compared to the PK film. The sample with the highest thermal resistance is PK-A. This shows that the anthocyanins and polyphenols in its structure interact strongly with the polymer matrix and stabilize the structure. In addition to the degradation start and end temperature values, there is also a significant difference in the T50 (temperature at which 50% of the degradation) value (Table 4).

#### 3.4.7. Thermal Transition Behavior of Films

DSC thermograms of the films are presented in Figure 8, while the corresponding glass transition (Tg) and melting (Tm) values are summarized in Table 4. The DSC thermograms displayed two characteristic transitions: a glass transition (Tg) between 37 and 73 °C and a melting endotherm (Tm) near 158 °C. The uniformity of Tm across all samples implies the preservation of PVA crystalline domains, while variations in melting peak intensities reflect differences in crystallinity. The neat PK film exhibited the highest Tg (~73 °C), indicative of a relatively rigid amorphous phase dominated by strong PVA–KGM interactions. The PK-A film, despite showing increased crystallinity via XRD, demonstrated a significantly reduced Tg (~43 °C), suggesting that Aronia anthocyanins acted as plasticizers in the amorphous regions by disrupting hydrogen bonds and enhancing chain mobility. Similarly, PK-D (~38 °C) and PK-AD (~37 °C) films showed even greater Tg reductions, indicating further amorphous flexibility imparted by betalains and/or combined phenolic extracts. In contrast, the PK-ADO film exhibited an intermediate Tg (~53 °C). This increase is plausibly due to the hydrophobic nature of thyme essential oil reducing water uptake, thereby diminishing water’s plasticizing effect on the amorphous phase. These observations resonate with literature findings: anthocyanin-rich natural extracts have been shown to reduce Tg by plasticization in PVA matrices (see, e.g., incorporation of anthocyanins into PVA/chitosan films) [89], while essential oils embedded within PVA-based films have also been reported to modulate Tg and crystallinity through complex interactions [89,90]. Meanwhile, the stable Tm across formulations confirms that crystalline domains of PVA are generally retained. Collectively, these results underscore how Tg shifts primarily reflect the mobility of the amorphous fraction, influenced by both bioactive plasticizers and moisture content adjustments, while Tm reflects the relative stability of semi-crystalline PVA structures.

#### 3.4.8. Moisture Barrier Properties: WVP and Moisture Content

The moisture content test expresses the tendency of samples to form hydrogen bonds with water molecules. PK-D film had higher moisture content than other films. This situation may have been caused by the hygroscopic properties of polyphenols in DFE or hydrophilic molecules (e.g., sugars, organic acids, etc.) in the extract. PK-AD exhibited similar moisture content values with PK-D. However, the addition of thyme EO reduced the moisture content due to the hydrophobic structure of the oil. The moisture contents of PK and PK-A films were statistically lower than other samples.

Food packaging acts as a protective barrier for the preservation of the product inside the packaging. The longer the two-way moisture transfer of the packaging, the more the product will be durable. The WVP values of the produced film samples are shown in Table 4. PK-A has the lowest WVP value. The polyphenol and anthocyanin content of AME causes tight molecular interactions in the polymer matrix, restricting the diffusion of water molecules. Similarly, in the study of Oun et al. [4], it was reported that the WVP value decreased in the aronia-added PVA/CS film compared to the control film and a denser film structure was formed. The PK-A film is followed by the PK-ADO film. The hydrophobic structure of the essential oil reduced the permeation of water. The amount of oil is important in the use of EO. It was stated that the barrier effect weakens in the KGM–TEO–PE film system developed by Liu et al. [81] if the high TEO content is not distributed homogeneously. There is no statistical difference between PK and PK-AD. However, the WVP values of these two films are low enough for packaging materials. The hydrophilic structures of DFE interacted with water and increased the WVP value of the PK film.

#### 3.4.9. Degradability of Films

The results of the 30-day degradation test conducted in a soil environment are presented in Table 4. All film samples exhibited certain levels of mass loss, indicating their biodegradable nature. The highest degradation rate was observed in the PK-D film containing the DFE additive. This result can be attributed to the fact that betacyanins are structures that are readily degradable in nature. The PK-D film was followed by PK-AD, PK-A, PK, and PK-ADO films, respectively. The reference film (PK) showed a moderate level of degradation under controlled conditions, which is associated with the inherent biodegradability of KGM present in the film structure. In contrast, the PK-ADO film, which contains thyme oil and a Pickering emulsion system, exhibited a lower degradation rate. This aligns with previous findings showing that thyme oil Pickering emulsions enhanced the protective performance of films [91] and that embedding essential oils in polymeric matrices reduced water accessibility and improved stability [92]. While films containing plant extracts tend to degrade more rapidly in soil, the presence of oil additives may delay the degradation process; however, this effect does not completely inhibit degradation. In this context, it can be concluded that all formulations are suitable for use as environmentally friendly packaging materials in terms of biodegradability.

#### 3.4.10. Antioxidant Activities of Films

**Figure 9:**
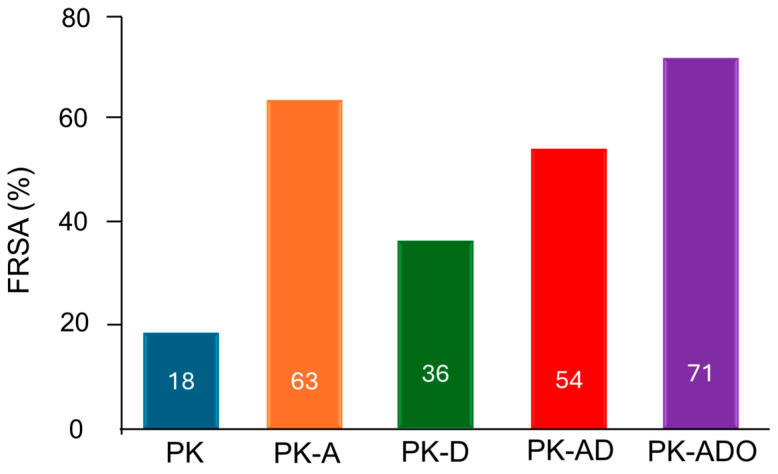
Antioxidant activities of biodegradable film formulations.

The radical scavenging capacities of the composite films were evaluated using the DPPH method (Figure 9). In the literature, the antioxidant activities of PVA and KGM were reported as 0.7% and approximately 4%, respectively [93,94]. Li et al. [80] reported the antioxidant capacity of the PVA/KGM reference film as 14.52 ± 3.01%. In our study, the reference film (PK) showed a limited antioxidant effect at 18% with its additive-free structure. This low value reveals that although PVA and KGM are structurally biodegradable, they cannot provide antioxidant effect due to the presence of hydroxyl groups and the absence of phenolic compounds. PK-A exhibited a significant radical scavenging activity at 63%. The high phenolic content of AME and the fact that it carries strong antioxidant compounds, especially anthocyanins, directly affected this result. This value obtained shows that the additive can maintain its antioxidant activity within the film matrix [34,42,63]. PK-D provided an antioxidant effect of 36%. Despite the low phenolic content of DFE, it is thought that this effect occurs due to betalain pigments, ascorbic acid and other phytochemicals with antioxidant properties. PK-AD showed a DPPH inhibition value of 71% in PK-ADO film and 54% in PK-AD film. This situation revealed the synergistic effect of extracts and extract + essential oil. Strong phenolic compounds such as carvacrol and thymol contained in thyme oil supported the increase in antioxidant capacity in the film. An important factor here is the PE agent that provides effective distribution of the oil in the water phase.

#### 3.4.11. Crystalline–Amorphous Phase Evolution

**Figure 10:**
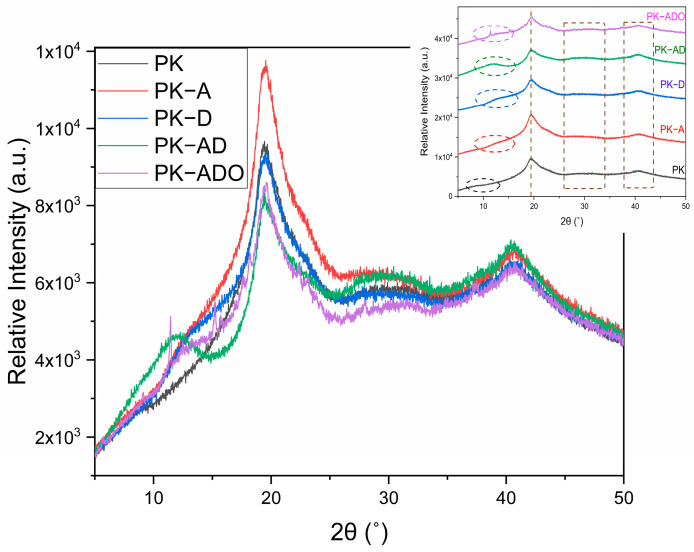
XRD patterns of neat PK and composite films (PK-A, PK-D, PK-AD, PK-ADO).

Figure 10 presents the XRD patterns of the neat and composite films, highlighting the crystalline–amorphous structures of the samples. The XRD pattern of the neat PK film exhibited reflections characteristic of both PVA and KGM. PVA, as a semi-crystalline polymer, typically presents four main crystalline peaks (2θ) at approximately 11.4°, 19.5–20.1°, 30.6°, and 40.8° [35,94]. In the neat PK film, diffraction signals appeared near ~10–11°, ~20°, ~30°, and ~40°, consistent with these reported positions. The strongest reflection at ~20° is attributed to the (101) plane of PVA, which overlaps with the broad amorphous halo of KGM between 20 and 22° [94]. This superimposition explains the broadened profile at ~20°, while weaker peaks at ~10–11°, ~30°, and ~40° confirm the semi-crystalline domains of PVA. Overall, the diffraction pattern of the PK film agrees with previously reported PVA/KGM blends, where PVA crystallinity is partially preserved but broadened by the amorphous contribution of KGM [35,94,95].

The PK-A film showed a distinct increase in the intensity of the ~20° peak with partial sharpening, indicating that phenolic and anthocyanin compounds interacted with hydroxyl groups of the matrix, promoting localized ordering in PVA domains. In contrast, the secondary reflections at ~10–11°, ~30°, and ~40° became less defined, reflecting disruption of long-range packing. Importantly, this structural change correlates with the mechanical results, as the PK-A film exhibited the highest tensile strength. The improvement can be attributed to enhanced chain packing around the ~20° region, a relationship consistent with earlier studies linking higher crystallinity to improved mechanical properties in PVA/polysaccharide composites [80].

The PK-D film presented a pattern highly similar to the neat PK film. The ~20° peak remained almost unchanged in intensity and position, indicating that the betalain-rich extract did not significantly affect the primary crystalline domains of the matrix. Only a slight reduction was observed around ~10–11°, suggesting minor amorphization. Higher-angle reflections at ~30° and ~40° were essentially unchanged. This result aligns with reports on starch/PVA films containing pitaya extracts, where low-to-moderate betalain levels caused only minor modifications in crystallinity [75].

The PK-AD film displayed a clear reduction in the intensity of the main ~20° peak compared to PK, PK-A, and PK-D. The simultaneous presence of anthocyanins and betalains disturbed molecular packing and reduced crystalline order. The ~10–11° reflection was further weakened, and the ~30° and ~40° peaks became barely discernible, indicating suppressed long-range order. These results are consistent with studies showing that combinations of polyphenolic and pigment-rich extracts tend to disrupt PVA chain rearrangement, leading to increased amorphous character [42,75].

The PK-ADO film exhibited the most pronounced amorphization. The main ~20° reflection was weakened and broadened, while the low- and high-angle peaks nearly disappeared. This strong suppression of crystallinity can be attributed to the plasticizing effect of thyme oil, which disturbs intermolecular interactions and crystalline packing in PVA. Similar amorphization upon essential oil incorporation has been reported in PVA-based films [35].

## 4. Conclusions

In this study, biodegradable films based on polyvinyl alcohol (PVA) and konjac glucomannan (KGM) were developed and functionalized with natural extracts (*Aronia melanocarpa*, dragon fruit) and thyme essential oil introduced via a Pickering emulsion system. The incorporation of these additives improved the films’ structural, barrier, thermal, and antioxidant properties, with noticeable differences depending on the type of extract used. In particular, Aronia extract enhanced strength and barrier performance, dragon fruit extract contributed flexibility and pH-sensitive coloration, while thyme oil increased hydrophobicity and opacity.

Overall, the results demonstrate that integrating polyphenolic extracts and essential oil emulsions into PVA/KGM matrices offers a sustainable and functional strategy for producing active and intelligent films. Such materials show promise for application in food packaging systems, where natural additives can contribute to both performance and consumer safety. Future studies are recommended to further assess microbial stability, shelf-life extension, and food interaction performance to complement the current findings.

## Figures and Tables

**Table 1:** Compositions of biocomposite films.

ID	PVA (% *v*/*v*)	KGM (% *v*/*v*)	Glycerol (% *v*/*v*)	Citric Acid (% *v*/*v*)	AME (% *w*/*v*)	DFE (% *w*/*v*)	β-CD (% *w*/*v*)	Thyme Oil (% *v*/*v*)
PK	90	10	4	5	-	-	-	-
PK-A	90	10	4	5	5	-	-	-
PK-D	90	10	4	5	-	5	-	-
PK-AD	90	10	4	5	2.5	2.5	-	-
PK-ADO	90	10	4	5	2.5	2.5	1	1

**Table 2:** Mechanical strength values of PVA/KGM films.

PVA/ KGM (*v*/*v*)	Elongation at Break * (%)	Young’s Modulus * (MPa)	Tensile Strength * (MPa)
90/10	538.69 ± 48.75 ^a^	9.02 ± 0.24 ^a^	13.97 ± 1.52 ^a^
75/25	714.01 ± 30.29 ^b^	6.96 ± 0.77 ^b^	9.64 ± 0.70 ^b^
50/50	380.00 ± 28.28 ^c^	0.86 ± 0.18 ^c^	1.89 ± 0.39 ^c^
10/90	455.67 ± 7.38 ^a,c^	0.77 ± 0.12 ^d^	1.23 ± 0.25 ^d^

* Different letters in the same column indicate significant difference (*p* < 0.05).

**Table 3:** Color parameter and mechanical strength values of all films.

Sample ID	L	a	b	ΔE	Opacity	Film Thickness * (mm)	Elongation at Break * (%)	Young’s Modulus * (MPa)	Tensile Strength * (MPa)
PK	96.64 ± 0.74 ^a*^	−0.22 ± 0.03 ^a^	0.86 ± 0.29 ^a^	3.16	3.67	0.43 ± 0.01 ^a^	538.69 ± 48.75 ^a,b^	9.02 ± 0.24 ^a^	13.97 ± 1.52 ^a^
PK-A	74.63 ± 5.34 ^b^	35.78 ± 6.65 ^b^	−5.54 ± 0.30 ^b^	43.95	4.83	0.42 ± 0.01 ^a,b^	382.71 ± 36.26 ^a^	15.87 ± 1.30 ^b^	17.56 ± 1.10 ^b^
PK-D	96.43 ± 0.20 ^a^	−0.65 ± 0.04 ^a^	4.92 ± 0.49 ^c^	6.13	4.04	0.50 ± 0.01 ^c^	619.49 ± 10.63 ^b^	11.72 ± 0.65 ^c^	14.92 ± 0.34 ^a^
PK-AD	81.81 ± 4.14 ^c^	24.77 ± 6.05 ^c^	−1.60 ± 0.31 ^d^	30.53	4.45	0.40 ± 0.01 ^b^	462.14 ± 36.22 ^a,b^	10.15 ± 0.95 ^a,c^	12.26 ± 0.94 ^a^
PK-ADO	74.09 ± 0.39 ^b^	28.30 ± 0.46 ^b,c^	2.29 ± 0.48 ^e^	38.19	5.02	0.44 ± 0.01 ^a^	600.37 ± 113.42 ^a,b^	11.55 ± 0.19 ^c^	14.72 ± 1.98 ^a,b^

* Different letters in the same column indicate significant difference (*p* < 0.05).

**Table 4:** Water vapor permeability, moisture content, water solubility and thermal values of biodegradeble films.

Sample ID	T50 (°C)	Tmax (°C)	Tg (°C)	Tm (°C)	WVP * × 10^−9^ (gm/m^2^sPa)	MC * (%)	SD (%)
PK	342	512	73	159	2.10 ± 0.27 ^c*^	10.98 ± 0.42 ^a^	35
PK-A	360	527	43	158	1.38 ± 0.09 ^d^	11.19 ± 1.25 ^a,b^	40
PK-D	344	532	39	158	2.78 ± 0.13 ^a^	15.28 ± 1.72 ^c^	52
PK-AD	347	552	37	159	2.15 ± 0.02 ^c^	13.62 ± 0.85 ^b,c^	48
PK-ADO	347	538	53	159	1.67 ± 0.21 ^b^	11.75 ± 0.79 ^a,b^	30

* Different letters in the same column indicate significant difference (*p* < 0.05).

## Data Availability

The data presented in this study are available on request from the corresponding author.
